# Do Commonly Used Measures of Pain Intensity Only Reflect Pain Intensity in Youths With Bothersome Pain and a Physical Disability?

**DOI:** 10.3389/fped.2019.00229

**Published:** 2019-06-20

**Authors:** Jordi Miró, Rocío de la Vega, Kevin J. Gertz, Ivan S. K. Thong, Mark P. Jensen, Joyce M. Engel

**Affiliations:** ^1^Unit for the Study and Treatment of Pain–ALGOS, Department of Psychology, Research Center for Behavior Assessment (CRAMC), Universitat Rovira i Virgili, Catalonia, Spain; ^2^Institut d'Investigació Sanitària Pere Virgili, Catalonia, Spain; ^3^Center for Child Health, Behavior and Development, Seattle Children's Research Institute, Seattle, WA, United States; ^4^Department of Rehabilitation Medicine, University of Washington, Seattle, WA, United States; ^5^Department of Psychology, National University of Singapore, Singapore, Singapore; ^6^Department of Occupational Science and Technology, University of Wisconsin-Milwaukee, Milwaukee, WI, United States

**Keywords:** pain assessment, pain intensity, psychosocial factors, physical disabilities, adolescents

## Abstract

The objective of this cross-sectional study was to evaluate the extent to which non-pain intensity factors influence the ratings of pain intensity on two commonly used measures: the Wong-Baker Faces pain rating scale (FACES) and the Verbal Rating Scale (VRS) in a sample of youths with physical disabilities and bothersome pain. Study participants came from a convenience sample of 115 youths (age: $\bar{X}$ = 14.4 years; SD = 3.3), who participated in a survey on the impact of pain in young people with a physical disability. They were administered measures of pain intensity, pain catastrophizing, depressive symptoms, pain interference, and pain control beliefs. Zero-order correlation analyses were used to examine the associations among the pain intensity scores, while regression analyses were used to test the influence of the non-pain intensity factors on the pain intensity scores. Although pain intensity scores from all scales were significantly associated with one another, the correlations were moderate. Regression analyses showed that the FACES and VRS also reflect pain interference, in addition to pain intensity. The fact that the FACES and VRS ratings reflect more than pain intensity should be considered when selecting a pain measure. The results of this study also provide information to help interpret results after treatment.

## Introduction

Research has shown that chronic pain is a major problem in individuals with disabilities (1, 2), including youths with disabilities (3–6). To identify the best treatments available for this population—and to develop better treatments—it is essential to have measures that provide valid and reliable information about pain. Although there are a variety of pain domains that are of interest (e.g., location, extension, frequency, duration), pain intensity is arguably the most important pain domain assessed by clinicians and researchers (7).

Some of the most commonly used self-report measures of pain intensity in youths are the 0–10 Numerical Rating Scale [NRS-11; e.g., (8, 9)], the Verbal Rating Scale [VRS; e.g., (10, 11)], the Wong–Baker FACES Pain Rating Scale [FACES; e.g., (12, 13)], and the revised form of the Faces Pain Scale [FPS-R; e.g., (14, 15)]. All these scales are known to have strengths and weaknesses; however, generally speaking, the NRS-11 has been recommended as the best option in most settings and with most populations (7, 8, 16).

The scores from these scales tend to be strongly related with each other. This strong relationship has been interpreted by researchers as indicating the measures provide the same information (17–19). However, there are also data showing that their scores are not completely concordant. For example, in one study (20) 126 schoolchildren between six and eight years of age were asked to report their pain intensity with four scales (i.e., a FPS-R, a NRS-11, a visual and a colored analog scales). Study results indicated that although these four scales measure one common factor, the measures were not consonant (i.e., their scores were not interchangeable).

Furthermore, in two recent studies, Jensen and colleagues have shown that the scores provided by these scales may reflect different domains, over and above pain intensity. In a study with a heterogeneous sample of 594 adults with different physical disabilities (including spinal cord injury, acquired amputation, neuromuscular disease, and multiple sclerosis) and chronic pain, Jensen et al. (21) found that the VRS pain intensity scores not only conveyed information about pain intensity, but also reflected patients' perceptions about pain interference and their beliefs about pain. Thong et al. (22) extended these findings by evaluating the influence of non-pain intensity factors on the scores of other commonly used pain intensity scales with a different sample of adult patients with chronic pain. They found that beyond pain intensity (as assessed by the NRS-11), VRS scores also reflected pain interference and FPS-R scores also reflected pain unpleasantness. Furthermore, they also found that the VAS and the NRS-11 scores were more strongly associated with each other than any other pairs of scale scores. As a group, these findings indicate that clinicians and researchers should not assume that the different self-report scales provide the same information.

However, the previous studies in this area were conducted with adults; it is not known if these results are generalizable to youths. This is important because some of these scales, such as the VRS and faces scales, are more likely to be used than others when working with young people. In this respect, a recent study has shown differences in how adults and youths designate pain intensity as mild, moderate or severe (23). Therefore, research evaluating the potential influence of non-pain intensity factors on the score of self-report questionnaires with samples of youths with pain is warranted.

Given these considerations, the aim of this research was to determine the extent to which psychosocial factors influence the ratings of pain intensity, in a sample of youths with physical disabilities and bothersome pain. Based on the results of recent studies, we hypothesized that ratings on the FACES and the VRS would be positively related with pain interference. In addition, we also hypothesized that ratings on these scales would show negative associations with cognitions thought to be “adaptive” (i.e., the belief that pain is a controllable experience), even when controlling for pain intensity. Regarding the extent to which depressive symptom severity and catastrophizing would contribute to the prediction of FACES and VRS ratings, we did not have a specific a priori hypothesis, as previous results regarding the roles that catastrophizing and depressive symptoms have as predictors of pain ratings have been inconsistent (21, 22).

## Materials and Methods

### Procedure

Data for this study and analyses came from a survey on the impact of pain in young people with a physical disability. A number of articles have been published using data from the original survey study, [e.g., (22, 23)], however none of these works examined the potential influence of psychosocial factors on pain intensity scores of the measures that were administered to participants.

The study procedures were approved by the Children's Hospital and Regional Medical Center's Institutional Review Board (Seattle, WA; USA). In order to participate in the primary study, youths had to: (1) have a diagnosis of neuromuscular disease, spina bifida, cerebral palsy, limb deficiency (acquired or congenital), or spinal cord injury; (2) be able to speak English; (3) have a chronological age from 8 to 21 years old; and (4) have no more than mild cognitive impairment (that is to say, having a Mini Mental State Examination [MMSE, (24)] score of 17/25 if administered in person or 15/22 if it was administered over the telephone. A modified MMSE for use with youths has demonstrated to provide reliable and valid scores when used with children as young as four years of age (25). In this group of participants, none had any cognitive impairment.

Participants that were 18 years of age or older provided their own assent or consent. For those younger than 18 years old, their parents or guardians gave written informed assent or consent to participate, and they also provided informed assent or written consent. When an in-person interview was not practically possible, interviews were conducted on the telephone.

**Figure 1:**
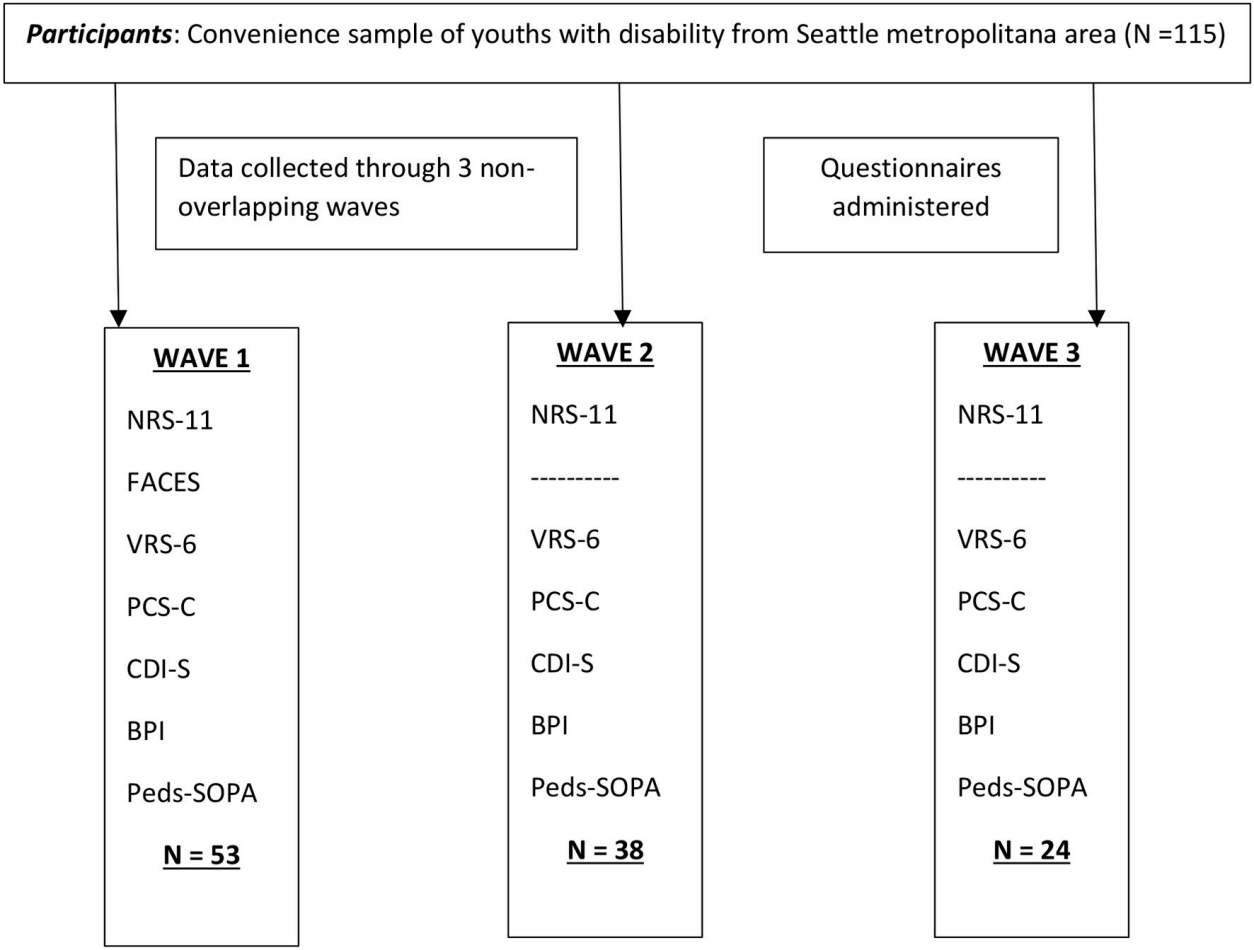
Flowchart.

Data collection occurred on three different occasions or waves, and not all participants were able to provide the same information (see Figure 1). The participants in each wave were non-overlapping; that is, all participants provided data just once. Important to this study, the FACES scale was administered only during the first wave of data collection (which was discontinued in order to reduce assessment load), therefore scores from this scale are just available for 53 participants. However, the Verbal Rating Scale and Numerical Rating Scale ratings were provided by all participants. All participants responded to the measures of pain catastrophizing, depressive symptoms, pain interference and pain attitudes.

### Measures

#### Average Pain Intensity

To report average pain intensity during the week prior to the interview, we used three different pain intensity scales: (1) the 0–10 Numerical Rating Scale (NRS-11), (2) the Wong-Baker FACES Pain Rating Scale [FACES; (26)], and (3) a 6-point categorical Verbal Rating Scale (VRS-6). With the NRS-11 participants are asked to select a number between 0 (“No pain”) and 10 (“Pain as bad as could be”) to rate their average pain intensity. Pain intensity reports provided with a NRS-11 have been found to be valid and reliable when used with youths (27) as young as 6 years of age (8, 28).

The FACES scale has six line drawings of faces that are meant to represent different levels of pain. A smiling face (described to respondents as a face that is: “Very happy because she/he does not hurt at all”) is scored as 0, and a face that is crying and appears very upset (described to respondents as a face of someone who: “…hurts as much as you can imagine, although you do not have to be crying to feel this bad”) is scored as 5 (26). The FACES was administered only during face-to-face interviews. This scale is commonly used to assess pain intensity in youths, and has demonstrated reliable and valid scores when used in this population (29, 30).

Participants were also asked to report their average pain intensity by choosing one of the six descriptors included in the VRS-6 (i.e., “None,” “Very mild,” “Mild,” “Moderate,” “Severe,” “Very severe”). Recent studies have shown that pain intensity ratings with a VRS are valid when used with youths with a physical disability (31).

#### Catastrophizing

We used the 13-item Pain Catastrophizing Scale for children [PCS-C; (32)] to measure pain-related catastrophizing. This scale requires responders to rate, on 5-point Likert scales, the extent to which they have catastrophizing thoughts when in pain. A total score is calculated by computing the responses to each item; the higher the score the greater the catastrophizing thoughts are in response to pain. Scores on the PCS-C have shown to have concurrent and discriminant validity, and high test-retest reliability over a 6-week period (33, 34). In this study sample, the internal consistency of the PCS-C was found to be good (α = 0.81).

#### Depressive Symptoms

We used the Children's Depression Inventory - Short Form [CDI-S; (35)] to assess depressive symptomatology. The CDI-S includes 10 items/symptoms that are presented as a series of three phrases. Respondents were asked to select the phrase that best represented how they felt (e.g., “Things bother me once in a while”/“Things bother me many times”/“Things bother me all the time”). Higher scores indicate more depressive symptomatology. The CDI-S has shown to provide valid and reliable scores when used with young people with a physical disability (36). In this study sample, the internal consistency of the CDI-S was found to be good (α = 0.82).

### Pain Interference

We used the Brief Pain Inventory [BPI; (37, 38)] to assess pain interference, which was slightly modified for use with this sample of individuals with a disability. The variations to the original questionnaire were: (1) adding three items relevant for this group of individuals that assessed pain interference with “*recreational activities*,” “*social activities*,” and “*self-care*,” which increased the content validity of the questionnaire; (2) revising the interference with “*walking*” item to allude to interference with “*mobility (*i.e., *the ability to get around)*” so that all participants could report on this particular issue—including those who are not able to ambulate independently; and (3) the item asking about “normal work” was changed to request about “*school, work, or chores*,” which was considered to be a more appropriate item age-wise. The BPI has been shown to provide valid and reliable scores when used with young people with a physical disability (39). In this study current sample, the internal consistency of the BPI was found to be excellent (α = 0.90).

### Pain Control Beliefs

We used items from the Control scale of the pediatric version of the Survey of Pain Attitudes [Peds-SOPA; (39)] to assess participants' beliefs about pain as a controllable experience. The Peds-SOPA has 29 true-false statements that can be scored to assess seven beliefs, with 6 of the items on the Control scale. Respondents are asked to rate how much they agree with each statement on a 3-point Likert scale (0 = “I do not agree with this,” 1 = “I am not sure,” 2 = “I agree with this”). The Peds-SOPA scale scores, including the Control scale, have been shown to be reliable and valid when used with youths with a physical disability (39, 40). In this study sample, the internal consistency of the Control scale was adequate (α = 0.72).

### Data Analysis

We first computed descriptive statistics for the demographic and study variables to describe the sample. We then evaluated the distributions of the study predictor variables as well as investigated potential multicollinearity of these variables to ensure they met the assumptions for the planned regression analyses. Next, we computed Pearson correlations between the three pain intensity measures to evaluate their zero-order associations. Finally, we conducted two hierarchical regression analyses to estimate the hypothesized associations between pain assessed with the FACES and the VRS, and measures of pain catastrophizing, depressive symptoms, and pain-related beliefs, after controlling for pain intensity as measured with the NRS-11.

In the hierarchical regression analyses, the FACES and VRS pain intensity ratings were the criterion variables. In the regression analysis predicting VRS ratings, we entered the NRS-11 ratings of average pain intensity in the first step, and sex in the second step as control variables. In the third step, the four independent variables (i.e., pain catastrophizing, depressive symptoms, pain interference and control beliefs) were entered as a block. In the regression analysis predicting FACES ratings, only three variables were entered in the third step. In this second analysis, depressive symptoms were not included because just half of the sample had been asked about and reported this information (see the Procedure section).

## Results

### Description of the Study Sample

Demographic and descriptive information for the 115 participants in the study is summarized in Tables 1, 2.

**Table 1:** Description of the study sample (*N* = 115).

**Variable**	**Percent**	***N***	**Mean (SD)**
Age, years		115	14.4 (3.3)
**SEX**			
Boys	56%	65	
Girls	44%	50	
ETHNICITY/RACE^*^			
Caucasian	68%	78	
Asian	11%	11	
African American	3%	3	
American Indian	3%	3	
Hispanic/ Chicano	4%	4	
Other	1%	1	
**DIAGNOSIS**			
Cerebral Palsy	34%	39	
Limb Deficiency	8%	9	
Spina Bifida	24%	27	
Muscular Dystrophy	25%	29	
Spinal Cord Injury	9%	11	

* *Race/ethnicity information was missing for 15 (13%) participants*.

*N, number of participants; SD, Standard Deviation*.

**Table 2:** Means and standard deviations of the study variables (*N* = 115).

	**Mean (SD)**
NRS-11 (0–10)	3.15 (2.43)
VRS (0–5)	3.06 (1.22)
FACES^*^ (0–5)	1.40 (1.06)
Pain Catastrophizing (PCS-C; 0-6)	2.19 (1.11)
Depressive Symptoms (CDI-S; 0-12)	2.51 (3.05)
Pain Interference (BPI; 0–10)	1.77 (1.77)
Control beliefs (Peds-SOPA; 0–4)	1.24 (0.45)

*NRS-11, 0–10 Numerical Rating Scale; VRS, Verbal Rating Scale; FACES, Wong-Baker FACES Pain Rating Scale; PCS-C, Pain Catastrophizing Scale for children; CDI-S, Children's Depression Inventory-Short Form; BPI, Brief Pain Inventory; Peds-SOPA, Pediatric version of the Survey of Pain Attitudes*.

*Range of scores for each questionnaire is provided in parentheses*.

* *N = 53*.

### Assumptions Testing

As shown by skewness and kurtosis statistics, the distributions of the study variables were adequately normal for the planned regression analyses (all were within the −2 and +2 range which is considered acceptable; skewness range = −0.19 to 1.49; kurtosis range = −0.95 to 1.61). In addition, all of the variance inflation factor values were below the standard cutoff value of 10 (ranging from 1.00 to 2.01), therefore showing that multicollinearity would not bias the findings from the regression analyses.

### Correlations Between Pain Intensity Scores on the Different Scales

The Pearson correlation coefficients between each pair of pain intensity ratings although on the lower range were significant, and stronger for those where the NRS-11 scores were involved. The correlation coefficients for the association between NRS-11 and FACES scores was 0.48 (*p* < 0.01) and the one between NRS-11 and VRS was 0.33 (*p* < 0.01), while the correlation coefficient for the association between FACES and VRS scores was 0.31 (*p* < 0.05).

### Linear Regression Analyses

#### Predicting VRS Ratings

The results of the regression analyses predicting VRS ratings are provided in Table 3. A direct positive effect was found for the NRS-11 pain intensity ratings as a predictor of the VRS scores in the first step (β = 0.30; *t* = 2.41, *p* < 0.05), accounting for 9% of the variance. In the second step, sex did not contribute significantly to the prediction of the VRS ratings. In the third step, only pain interference was statistically significant (β = 0.36; *t* = 2.15, *p* < 0.05), accounting for an additional 17% of the variance. Catastrophic thinking about pain, control beliefs, and depressive symptoms did not contribute significantly to the prediction of the VRS ratings.

**Table 3:** Results of the linear regression analyses (*N* = 115).

**Step**	***R^2^***	**R^2^ change**	***F* change**	**B to enter**	***t*-value**
**CRITERION VARIABLE: VERBAL RATING SCALE**					
1. Pain intensity (NRS-11)	0.09	0.09	5.81^*^	0.30	2.41^*^
2. Sex	0.09	0.00	0.02	0.02	0.16
3. Pain catastrophizing	0.26	0.17	2.92^*^	0.11	0.80
Control beliefs				−0.11	−0.86
Depressive symptoms				0.09	0.66
Pain interference				0.36	2.15^*^
**CRITERION VARIABLE: FACES**^**#**^					
1. Pain intensity (NRS-11)	0.23	0.23	14.76^**^	0.47	3.84^**^
2. Sex	0.24	0.01	0.75	−0.11	−0.87
3. Pain catastrophizing	0.35	0.11	2.60^*^	0.12	0.89
Control beliefs				0.49	0.38
Pain interference				0.41	2.42^*^

*NRS-11, 0–10 Numerical Rating Scale; FACES, Wong-Baker FACES Pain Rating Scale*.

* p < 0.05;

** *p < 0.001*.

# *N = 53*.

#### Predicting FACES Ratings

As can be seen in Table 3, the findings show a direct positive effect of pain NRS-11 pain intensity ratings on the FACES scores (β = 0.47; *t* = 3.84, *p* < 0.001), accounting for 23% of the variance. In the second step, sex did not contribute significantly to explain the variance in FACES. In the third step, only pain interference made a statistically significant (β = 0.41; *t* = 2.42, *p* < 0.05) and independent contribution to the prediction of the FACES ratings, accounting for an additional 13% of the variance. Neither catastrophic thinking about pain nor control beliefs made a statistically significant additional contribution to the prediction of the FACES ratings.

## Discussion

This study provides new information on the potential influence of psychosocial factors on pain intensity reports, in a new sample of youths with a physical disability and bothersome pain. As hypothesized, pain intensity ratings provided with the VRS-6 and the FACES were influenced by psychosocial factors, as demonstrated by their significant associations with a measure of pain interference. Therefore, when a youth identifies her or his pain as “very severe” or selects the facial expression indicating the most severe pain using the FACES scale, she or he may not *only* be describing the intensity of the experienced pain, but also that the pain is interfering in her or his life.

However, inconsistent with the study hypotheses, the influence of pain cognitions on pain ratings, was not supported. These results differ from those reported by Jensen et al. (21), which, in a study with a sample of adults with a disability and chronic pain found that VRS ratings were associated with both positive and negative pain beliefs. Nevertheless, the results from the present study are consistent with the results reported by Thong et al. (22), who also did not find an association with maladaptive cognitions. The discrepancy in these findings might be related to the different measures used to assess pain catastrophizing. That is, while Jensen and colleagues used the 6-item Catastrophizing Scale of the Coping Strategies Questionnaire (41), in this work and in Thongs and colleagues' study the Pain Catastrophizing Scale (42) was used. Furthermore, Jensen and colleagues used a composite score containing information about pain catastrophizing, while in this study and the one conducted by Thong and colleagues measured pain catastrophizing directly. The null findings with respect to the FACES scale may also be related to a potential lack of power for identifying predictors of the FACES scale ratings in this sample, due to the lower number of participants who completed this scale. The inconsistency of the findings indicates that additional studies are warranted to help understand the extent to which, and under what circumstances, pain cognitions may influence pain intensity ratings.

Regarding the influence of depressive symptom severity on pain intensity ratings, the results were again in line with those from Thong et al. (22), showing no significant association between pain intensity ratings as reported on the VRS-6 and the severity of depressive symptoms. However, we could not study the potential influence of depression on the FACES pain intensity ratings due to the small number of participants (*N* = 53) that had responded to both of these measures in the sample. Previous studies have suggested that faces scales like the FACES can be viewed as not only providing information about pain intensity but also about emotional responses to pain, because their use of smiling faces and a face with tears (31, 43). Therefore, additional research is needed to clarify the potential influence of emotions on these types of scales as it seems in general that youths prefer to use facial scales in comparison to other scales like the numerical rating scale (44, 45).

A finding emerged from these analyses that bears discussion; that is, the associations among each pair of intensity measures was only moderate. Previous research has shown that such associations tend to be much stronger. Most studies report correlations that are in the 0.70s or higher [e.g., (27), (46–48)]. Nevertheless, sometimes these correlations are a little bit lower. For example, a recent study by Ruskin et al. (49) reported lower correlation values between scores on a NRS-11 and a Colored Analog Scale (CAS; *r* = 0.58) when measuring current, usual, or strongest pain intensity.

Therefore, in new populations, such as youths with a disability and chronic/bothersome pain who participated in the current study, and for whom the measures have not yet been validated, it might be useful to ask about pain intensity using multiple scales as a way to “check” the validity in that particular sample/population. Then, if weak, a composite score may be most appropriate. Nevertheless, the moderate associations found among the measures used in this study and in this population of youths with physical disabilities indicates that additional studies are warranted to help decide the best approach for assessing pain intensity.

There are some limitations to this study that should be taken into account. First, the sample was one of convenience. Therefore, the generalizability of the findings to other samples of youths with a disability remains to be clarified. Thus, it would be important to replicate the study with other populations (e.g., youths with cognitive impairments such as in autism, youths with chronic pain as a primary presentation such as in abdominal pain, or youths with chronic pain as a secondary problem such as in juvenile idiopathic arthritis), and with other commonly used scales [e.g., the Faces Pain Scale—Revised; (14)]. Second, even though the number of participants could be considered large, compared to other studies in this area, the sample size was relatively small, in particular in relation to the analysis of the FACES scale. Thus, research with larger sample sizes, in particular, research with larger sample sizes that are administered FACES scales, is needed to help determine the reliability of the findings.

Despite the study's limitations, the findings help to advance our knowledge regarding the meaning of pain intensity ratings in young people with physical disabilities. Studies like this one provide additional important information to help decide what scale is best to use and how to interpret results after treatment. For example, the results in this study show that changes in pain intensity scores as provided with a verbal rating scale and the FACES, may also mean changes in domains other than pain intensity, particularly in pain interference. Therefore, clinicians and researchers should take into account this information when interpreting the results of their interventions and studies in which the VRS-6 and FACES are used.

## Ethics Statement

This study was carried out in accordance with the recommendations of the Children's Hospital and Regional Medical Center's Institutional Review Board, with written informed consent from all subjects. All subjects gave written informed consent in accordance with the Declaration of Helsinki. The protocol was approved by the Human Subjects Review Committee.

## Author Contributions

JM and MJ designed and developed the idea for the study. JE, KG, and MJ contributed to the collection of the data. RdlV and JM developed the statistical analysis. All authors contributed to the analysis of the results. JM wrote the first version of the manuscript. All authors contributed to the different versions of the manuscript and all agreed to this submission.

### Conflict of Interest Statement

The authors declare that the research was conducted in the absence of any commercial or financial relationships that could be construed as a potential conflict of interest.
